# Changes in Functional Outcomes After an Inpatient Rehabilitation Program for Solid-Organ Transplant Recipients

**DOI:** 10.1177/15269248231189861

**Published:** 2023-07-25

**Authors:** Matheus de Paiva Azevedo, Patrícia Angelica de Miranda Silva Nogueira, Lorna D’Souza, Betty Cheung, Karen Uy, John Patcai, Sunita Mathur, Tania Janaudis-Ferreira

**Affiliations:** 1 School of Physical and Occupational Therapy, 5620 McGill University, Montreal, QC, Canada; 2 Departamento de Fisioterapia, 28123 Universidade Federal do Rio Grande do Norte, Natal, Brazil; 3 142153 St John's Rehab Program, Sunnybrook Health Sciences Centre, Toronto, ON, Canada; 4 Departments of Medicine, 7938 University of Toronto, Toronto, ON, Canada; 5 School of Rehabilitation Therapy, 4257 Queen's University, Kingston, ON, Canada; 6 Respiratory Epidemiology and Clinical Research Unit, Centre for Outcomes Research and Evaluation, Research Institute of the McGill University Health Centre, Montreal, QC, Canada; 7 Canadian Donation and Transplantation Research Program, Li Ka Shing Centre for Health Research Innovation University of Alberta, Edmonton, AB, Canada

**Keywords:** clinical outcomes, exercise outcomes, quality of life, research, quantitative methods, correlational, solid organ transplantation, inpatient rehabilitation

## Abstract

**Introduction:** Outpatient exercise training has been shown to be beneficial for solid organ transplant recipients. Little is known about the effects of inpatient rehabilitation programs for recipients with a more complicated postoperative course. **Research Question:** This study was designed to (1) describe the changes in functional outcomes after an inpatient rehabilitation program, and (2) determine whether the changes in lower body strength and quadriceps strength are associated with changes in functional exercise capacity. **Design:** This was a single-arm prospective longitudinal study. The recipients participated in an inpatient rehabilitation program twice a day, 7 days a week for 3 to 4 weeks. **Outcome Measures Included:** 2-Minute Walking Test, Timed Up and Go, Berg Balance Scale, 30-Second Sit to Stand, biceps and quadriceps strength, Functional Independence Measure, SF-36, and Canadian Occupational Performance Measure. **Results:** Twenty-eight patients (54% female, mean age = 55 [11]) completed the study. Participants were mostly liver (42%) and lung recipients (35%). There were statistically significant improvements in all outcomes after the intervention. There was no relationship between changes in functional exercise capacity and quadriceps strength or lower body strength. **Conclusion:** An inpatient rehabilitation program may improve several functional outcomes and health-related quality of life in transplant recipients with a complicated postoperative course.

## Introduction

Solid-organ transplantation is one of the major medical advances of the 20th century. It is proven to decrease mortality and improve the quality of life of people living with end-stage liver, heart, kidney, pancreas, or lung conditions.^1^ Pretransplant factors such as deconditioning, anemia, nutritional depletion, and depression as well as posttransplant factors such as effects of immunosuppressant medications, respiratory failure, prolonged hospitalization, myopathies, neuropathies, and episodes of organ rejections can negatively impact recipients’ health and physical function.^2^ Poor physical function in recipients can be associated with rehospitalizations,^3^ poor health-related quality of life (HRQoL), and increased mortality.^4^

The benefits of exercise training in transplantation are well established.^2,5^ It improves HRQoL, physical function, maximal exercise capacity, quadriceps muscle strength, and diastolic blood pressure.^5^ However, most of the published studies have focused on recipients who were independent and able to participate in an outpatient program.^5^ There is limited research on recipients with a more complicated course who need additional care and may be referred to a specialized inpatient rehabilitation program. The few existing studies examining the effects of inpatient rehabilitation for recipients have shown that this type of program is feasible, safe, and well-tolerated by patients.^6–8^ These studies have also shown that an inpatient rehabilitation program for recipients may improve physical function, muscle strength,^7^ functional exercise capacity, activities of daily living, and independence.^6,8^ In addition to the clinical benefits, an inpatient rehabilitation program may impact readmission to hospitals and reduce healthcare utilization.^9^

A retrospective study with over 3000 patients examined the association between different discharge locations post-transplant and 30-day readmissions to the hospital and observed that recipients who were discharged to an inpatient rehabilitation program had less risk for readmission within 30 days posttransplant.^9^ Despite the preliminary evidence for the effectiveness of an inpatient rehabilitation program for recipients, most of the studies on inpatient rehabilitation focused on a single organ group and were either case studies or retrospective studies that consequently had limited control over the choice of outcome measures and how they were measured.

Lower limb muscle strength has been shown to be associated with decreased exercise and functional capacity of individuals with chronic conditions.^10^ Considering that the main goal of inpatient rehabilitation is to improve patients’ independence, understanding the relationship between lower body strength and functional exercise capacity is critical to planning effective interventions to improve walking and consequently independence.

The aims of this study were (1) to describe the changes in functional outcomes after an inpatient rehabilitation program for solid organ transplant recipients, and (2) to determine whether the changes in lower body strength and quadriceps strength are associated with changes in functional exercise capacity.

## Methods

### Study Design and Data Collection Procedures

This was a single-arm prospective longitudinal study conducted at St John's Rehab (SJR) Program of the Sunnybrook Health Sciences Centre in Toronto, Canada. The study was approved by the Research Ethics Board. The inpatient rehabilitation program is a dedicated program for transplant recipients.^11^ After obtaining informed consent, patients were required to attend 2 pre and 2 postintervention assessment sessions. The preintervention assessment sessions occurred within the first 2 days of admission. The postintervention assessment sessions occurred within 1 or 2 days of hospital discharge. The first preintervention assessment session was conducted by the physiotherapist of the inpatient rehabilitation program. Patients were asked to perform the Two-Minute Walk Test (2MWT), the Berg Balance Scale (BBS), and the Timed Up and Go (TUG). A research coordinator was responsible for the second pre-intervention assessment that included a second 2MWT, muscle strength testing, the 30-second Sit-to-Stand test (30-STS), the Canadian Occupational Performance Measure (COPM), and the SF-36 Health Survey. During the second assessment session, measurements were collected of anthropometry, demographics, type of transplant, length of acute hospital stay, and functional independence measure (FIM). Once the outcome measures were collected at the preintervention assessment sessions, patients commenced the multidisciplinary inpatient rehabilitation program. The outcome measures were again collected after the rehabilitation program over 2 different days. The same assessors administered the same tests or questionnaires postintervention.

### Population

The Multi-Organ Transplant Program of the Toronto General Hospital performs approximately 621 solid organ transplants per year. In 2022, 37% of the recipients were female and the mean age of all recipients was 55.4.

### Sampling

Recipients of liver, kidney, heart, lung, and pancreas transplants who were referred from the Multi-Organ Transplant Program of the Toronto General Hospital to a multidisciplinary inpatient rehabilitation program from August 2012 to March 2014. The Toronto General Hospital referred patients to the inpatient rehabilitation program if they had problems with body functions, structures, activities, and participation.^11^ The most common problems that patients experienced were extreme fatigue, severe deconditioning, nutritional depletion, metabolic toxicity, myopathy, neuropathy, cognitive loss secondary to the disease or its treatment, and depression. Medical complications could include rejection of the transplanted organ, anemia, cardiac failure, respiratory failure, and infection.^11^

The exclusion criteria for the study were (a) inability to understand English; (b) known cognitive impairment; (c) evidence of a neurological or musculoskeletal condition that could severely limit mobility and (d) transplantation not being performed within 6 months of inpatient rehabilitation admission.

### Intervention

The inpatient rehabilitation program is a multidisciplinary program that comprises Physical Therapy, Occupational Therapy, Nursing, Psychology, Physiatry, Hospitalist Physician, and nutritional support.^11^ Discharge from the program occurs when the initial established goals for rehabilitation are reached and if discharge home is deemed safe by the healthcare team. Necessary equipment and follow-up services and appointments need to be arranged before discharge. The physical therapy program was offered twice a day, 7 days a week for 3 to 4 weeks.^11^ The physical therapy exercises were individualized, tailored to each patient's functional abilities, and consisted of (a) supervised mobility/transfer exercises; (b) range of motion and resistance exercises (bridging, toe/heel, hip flexion, hip abduction, hip extension, hamstring curls, and mini squats or sit to stand); (c) endurance exercises (marching on the spot, or recumbent bicycle or treadmill) and (d) balance training. All exercises were individualized based on the patient's baseline assessment and each session lasted about 45 min.

### Data Collection

#### Primary Outcome Measure

##### Two-Minute Walk Test

The 2MWT is a valid and reliable test that measures functional exercise capacity in different clinical populations.^12^ The test was administered in a corridor of approximately 30 m and supervised by a clinician and research assistant. Patients were instructed to walk as far as they could in 2 min and standardized instructions were given every 30 s. Two 2MWT were performed on 2 different days to account for learning effects. Individuals were allowed to use their gait aid if required. The highest walked distance in meters was used for analysis. The 2MWT was chosen over the 6MWT as the 2MWT was routinely used in the program and because the 6MWT can be burdensome for patients with higher disability levels.^12^

#### Secondary Outcome Measures

##### The Berg Balance Scale

The BBS is a 14-item performance-based instrument that measures functional balance and that can be used to assess response to treatments.^13^ It includes items such as single-legged stance, transfers, reaching, and turning around. Each item consists of a 5-point ordinal scale ranging from 0 to 4, with 0 indicating the lowest level of function and 4 the highest level of function. The maximum score is 56. Scores below 45 points indicate a higher risk of falling. The BBS has been shown to be responsive to an inpatient rehabilitation program in a sample of heart and lung recipients.^8^

##### The Timed Up and Go

The TUG is a psychometrically robust test that was designed to evaluate the risk of falls by observing the patient's postural stability, gait, stride length, and sway.^14^ The test requires the patient to rise from a standard armchair, walk 3 m at a comfortable and safe pace, walk back to the chair and sit down. The time to complete the test is presented in seconds. After a practice test, the second trial was recorded on the same day and the best results would be used for analysis. Individuals were allowed to use their gait aid if required.

##### The 30-Second Sit-to-Stand Test

The 30-STS test is a valid and reliable test to assess lower body strength in older adults.^15^ The test requires the patient to cross their arm across their chest and stand up and sit down as many times as possible in 30 s. Results were recorded in the number of repetitions completed.

##### Peripheral Muscle Force

Peripheral muscle force was assessed using an isometric hand-held dynamometer (MicroFET 2; Hoggan Scientific, Salt Lake City, UT). Measurements of elbow flexion and knee extension were performed on the dominant side and results were reported in Newtons. The test was performed with patients in a sitting position. The average of the highest 3 measures within 5% of each other were used for analysis. Good reproducibility of these tests has been previously shown.^16^ In addition, the research assistant practiced the assessment with 10 healthy individuals before initiating the study to ensure good intrarater reliability. Elbow flexion and knee extension force are associated with performance in upper and lower limb functional tests and independence in activities of daily living.^17^

##### Functional Independence Measure

The FIM is a disability scale widely used in rehabilitation settings. It has been shown to be reliable and valid as a measure of assessment of functional independence.^18^ The scale contains 18 items each scored from 1 (totally dependent) to 7 (fully independent). For this study, the FIM motor subscales were used, which included the areas of self-care (eating, grooming, bathing, upper-body dressing, lower-body dressing, toileting), sphincter control, mobility, and locomotion.

##### The Canadian Occupational Performance Measure

Problematic activities of daily living (ADL) were identified using the COPM. It is an individualized, patient-centered measure designed to identify participants’ self-perception of occupational performance and can be used with individuals with a variety of disabilities.^19^ It includes questions regarding self-care, productivity, and leisure. Scores were calculated by summing individual problem scores and dividing them by the number of problems.

##### SF-36 Health Survey

The SF-36 is a generic measure and short-form health survey with 36 questions. It assesses functional health and well-being from the patient's perspective and reports on eight domains. It is a practical, reliable, and valid measure of physical and mental health that has been tested among kidney transplant recipients previously.^20^ Each domain is transformed to a 0-100 scale on the assumption that each question carries equal weight. Lower scores indicate a lower HRQoL.

### Data Analysis and Sample Size Calculation

The within-subject difference in the 2MWT following rehabilitation represents the primary outcome. To calculate the sample size, preliminary data were collected from 10 recipients that had participated in the inpatient rehabilitation program. The data showed that the mean change in 2MWT after rehabilitation was 23.46 (16.36) meters. Using an online calculator, it was determined that 6 patients would be required to yield 80% power (α = .05) to detect a mean difference of at least 23 m in the 2MWT using a two-tailed paired *t*-test and SD of 16.36. However, the aim was to include 30 patients for several reasons: to account for 20% of dropouts, be within a range of sample size suggested for rehabilitation studies for power above 80% and to be within the study budget.^21^

Statistical analyses were performed using SPSS v 26 (IBM Corp. Armonk, NY). All variables were tested for normality. To address the first objective, a student two-tailed paired *t*-test was used for normally distributed variables and Wilcoxon for nonnormally distributed. To address the second objective, we used the Pearson correlation coefficient to estimate the associations between the scores of the 30-STS and quadriceps strength with the 2MWT. A *P*-value ≤ .05 was considered significant. Data from all patients recruited to participate in the study were analyzed irrespective of their attendance or compliance with the rehabilitation program.

## Results

**Figure 1.**
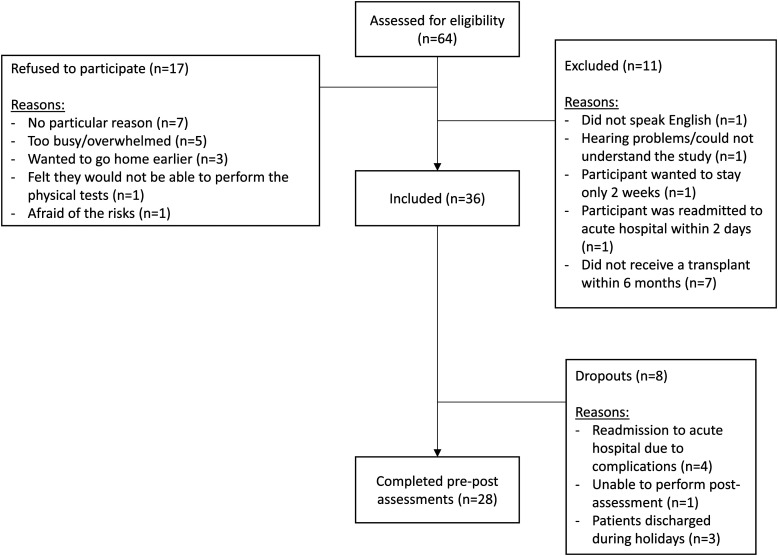
Flowchart of participants to be included in the inpatient rehabilitation intervention study.

During the study period of 1 year and 4 months, 64 patients were approached, 17 patients declined to participate in the study, and 11 did not meet the eligibility criteria. Thirty-six recipients agreed to participate in the study, 8 dropped out and 28 completed the study. A detailed flowchart of the study and reasons for dropouts can be found in **Figure 1**. Four participants were re-admitted to the acute care facility due to gastrointestinal and hemodynamic complications but were able to return to the program within 4 days and, therefore, completed the study. The characteristics of the patients are presented in **Table 1**.

**Table 1.** Characteristics of the Participants.

Characteristics (N = 28)	Mean (SD)
Age	55 (11)
BMI	25 (4)
Days in acute care before transplant	53 (38)
Days in acute care after transplant	56 (45)
Days in rehabilitation program	22 (7)
	N (%)
Females	15 (54%)
Type of transplantation	
Liver	12 (43%)
Lung	12 (43%)
Heart	2 (8%)
Liver and kidney	1 (3%)
Kidney and pancreas	1 (3%)

Abbreviation: BMI, body mass index.

All functional outcomes improved significantly post-intervention (*P *< .001, CI 95%). The mean changes of each outcome can be found in **Table 2**. Nine patients required the use of a rollator to perform the 2MWT and the TUG in the pre-and post-intervention assessments. Only 2 patients were able to perform the 30-STS pre-intervention while 8 were able to perform it post-intervention. The mean missing data for each variable was 3.5% (1 participant).

**Table 2.** Changes in Functional Outcomes Postintervention (N = 28).

Outcomes	Pre: Mean (SD)	Post: Mean (SD)	Mean change (SD)	95% CI
2MWT (meters)	69 (34)	100 (35)	31 (19.2)	23.9-38.1
30-STS (repetitions)	0.35 (1.2)	1.31 (2.4)	1 (2)	0.2-1.7
Biceps strength (Newton)	21 (8)	25 (9)	4 (3.3)	2.7-5.2
Quadriceps strength (Newton)	20 (7)	26 (8)	6.3 (4.1)	4.7-7.8
SF-36 total (points)	39 (14)	56 (16)	17 (13.4)	12-22
BBS (points)	32 (12)	45 (7)	13 (8.8)	9.7-16.3
FIM (points)	54 (12)	74 (8)	20 (13.6)	15-25
TUG (seconds)	23 (12)	16 (9)	−7 (6.6)	−9.4 to −4.5
COPM performance (points)	3 (1.6)	6 (2.1)	3 (1.9)	2.3-3.7
COPM satisfaction(points)	3 (1.8)	6 (2.3)	3 (1.9)	2.3-3.7

Abbreviations: 2MWT, two-minute walk test; 30-STS, 30 s sit-to-stand; SF-36, short-form 36 items Health-Related Quality of Life (total score; lower scores represent lower HRQoL); BBS, Berg Balance Scale (lower scores represent a higher risk of fall and worse balance); FIM, Functional Independence Measure (lower scores represent less independence); TUG, Timed Up and Go; COPM, Canadian Occupational Performance Measure (lower scores represent poor performance and low satisfaction).

Pearson coefficients of correlation between the changes in 2MWT and 30-STS demonstrated no association (*r* = .102). A similar result was found between changes in 2MWT and quadriceps strength (*r* = .127). There were no statistically significant differences in mean changes of any outcome when participants of each organ type were compared, except in the performance subscale of the COPM.

## Discussion

This study prospectively and comprehensively examined the changes in functional outcomes after an inpatient rehabilitation program in different types of transplant recipients. Recipients who were referred to an inpatient rehabilitation program before being discharged home following transplantation improved their functional exercise capacity, mobility, balance, lower body strength, quadriceps, and biceps muscle strength, ability to perform daily activities, and health-related quality of life. No associations were found between changes in functional exercise capacity and changes in lower body strength or quadriceps strength.

The findings were consistent with other inpatient rehabilitation studies of specific organ groups. Shiner et al^8^ have also shown an improvement in functional exercise capacity (as measured by the 6MWT), BBS, and TUG scores after an inpatient program among heart and lung recipients. Patcai et al^11^ have shown improvements in FIM after an inpatient rehabilitation program in solid-organ transplant recipients.

In contrast to these findings, Ihle et al^22^ in a 2011 study that evaluated the effects of inpatient rehabilitation in lung transplant recipients, found no difference in HRQoL between intervention and control groups. Considering the lack of a comparison group in this study, no confirmation of changes in the HRQoL and physical function were a result of the inpatient program or natural recovery after transplant. Longitudinal studies (where rehabilitation was not offered) have shown natural recovery in functional outcomes and HRQoL in recipients at 6- and 12-months posttransplant. The postintervention assessment in this study occurred earlier (within 2-3 months posttransplant); therefore, due to the magnitude of change found, the improvements seen were likely to be a result of the rehabilitation program rather than natural recovery. For example, the mean change in the primary outcome (2MWT), which was assessed early posttransplant (within 2-3 months), was 3 times higher (31 [19.2] m) than the minimal detectable change in older adults (9.1 m).^12^ In a previous study where an exercise program was offered to lung transplant candidates, the mean change between the six-minute walk distance at a time for assessment for transplant and 1-month posttransplant was 37 m, which was slightly above the minimally important difference for adults with chronic lung disease (30 m).^23^

The 30-STS or dynamometry has not been used to assess lower limb strength or biceps or quadriceps muscle strength, respectively, in transplant recipients participating in an inpatient rehabilitation program. However, Tarrant et al^24^ used the 60-second sit-to-stand test in a sample of lung recipients in the acute care setting and found a mean of 13 repetitions. This confirmed how severely impaired the sample in this study was (mean 30-STS at baseline = 0.35 repetitions). This severe impairment in lower limb muscle strength may be partly explained by the long length of stay in the hospital posttransplant (mean 56 days). The median length of stay in the inpatient ward in the study by Tarrant et al^24^ was 7 days for medical readmission patients and 19 days for immediate postoperative lung transplant recipients.

The mean change in the 30-STS in this study was only 1 repetition. In a recent study conducted among older adults undergoing rehabilitation, the minimal detectable change for the 30-STS was found to be 0.70, which suggests that an additional repetition was likely not a measurement error and may represent a real functional improvement.^25^ The improvements in lower body strength and biceps and quadriceps strength found were expected as the exercise training included resistance exercises. After the intervention, the number of patients who were able to perform the 30-STS test was 4 times higher. This increase is relevant as this was an important functional ability to prevent falls.^15^

No correlations were found between the 2MWT and quadriceps strength (*r* = .127, *P *< .02) or 30-STS (0.102, *P *< .03). A study from 2015 by Kjølhede et al^26^ found a moderate positive correlation between 2MWT scores and maximal muscle strength measured by an isokinetic dynamometer in people with multiple sclerosis. In 2018, Bokaeian et al^27^ found a weak positive correlation between quadriceps strength and the 2MWT among patients with knee osteoarthritis. In this study, an isokinetic dynamometer to measure quadriceps strength was not used, which makes it difficult to compare the findings with these previous studies. Studies with a larger sample size are needed to confirm whether impairments in lower body muscle strength impact functional exercise capacity in transplant recipients.

Most of the dropouts were due to clinical complications such as respiratory failure and hemodynamic instability. Patcai et al^11^ studied a similar cohort of recipients in the same rehabilitation program and observed that these complications occurred within 3 days of admission to the inpatient rehabilitation program. None of the reasons for readmission to acute care were related to the rehabilitation program, which was consistent with previous studies that demonstrated that an inpatient program can be considered safe among recipients.^7–9^

The study had some strengths and limitations. One of the strengths was that changes in recipients were prospectively and comprehensively evaluated for functional outcomes and HRQoL after an inpatient rehabilitation program. The improvements occurred in all transplant types, making it a relevant and successful program. This type of program enables recipients to improve their functional ability^6–9,11^ and it may reduce complications as well as decrease healthcare costs by reducing the risk of readmission.^9,11^ Further cost savings may be realized by transferring the patient to a cost-effective rehabilitation program before they could possibly be discharged home, thus saving valuable acute transplant program bed days.^11^

A limitation was the lack of a control group, which makes it difficult to determine if the improvements in the outcomes were due to the natural recovery posttransplant or because of the inpatient rehabilitation program. In addition, since the program was multidisciplinary, different components of the program (eg, physical therapy, occupational therapy, and nutrition) may have had an impact on the improvements of physical function in the participants; however, no determination of the contribution of each component was assessed. Forty-four percent of the approached patients did not want to participate or were excluded from the study. There was no data on these patients; however, the reasons for declining participation and for exclusion were recorded. Most of the excluded patients had had their transplant between 2 and 4 years prior to their admission to inpatient rehabilitation. These patients were not referred by the Toronto General Hospital like other patients with recent transplants. They were admitted to the program for other reasons than issues related to their transplant (eg, a fall at home) and because they had already been a patient in the program early posttransplant.

Although the data were collected between 2012 and 2013, the results of the study remain relevant as the program and criteria for referral remain the same. There is limited availability of such programs for transplant recipients worldwide and these findings may influence the development of new programs. Finally, as a single-centered study, generalization bias is expected, but with the lack of inpatient rehabilitation programs in Canada, multi-center sampling was impossible.

## Conclusions

A multidisciplinary inpatient rehabilitation program may improve several functional outcomes and health-related quality of life among transplant recipients who have experienced a more complicated course of transplantation. The findings of this study may encourage patients and healthcare professionals working in transplantation to advocate for more inpatient rehabilitation programs for recipients who are not able to be discharged home following transplantation.
